# Comparison of metagenomic and targeted methods for sequencing human pathogenic viruses from wastewater

**DOI:** 10.1128/mbio.01468-23

**Published:** 2023-10-25

**Authors:** Harry T. Child, George Airey, Daniel M. Maloney, Abby Parker, Jonathan Wild, Suzie McGinley, Nicholas Evens, Jonathan Porter, Kate Templeton, Steve Paterson, Ronny van Aerle, Matthew J. Wade, Aaron R. Jeffries, Irene Bassano

**Affiliations:** 1 Biosciences, Faculty of Health and Life Sciences, University of Exeter, Exeter, United Kingdom; 2 Institute of Infection, Veterinary and Ecological Sciences, University of Liverpool, Liverpool, United Kingdom; 3 Institute of Ecology and Evolution, University of Edinburgh, Edinburgh, United Kingdom; 4 Viral Genotyping Reference Laboratory Edinburgh, NHS Lothian, Royal Infirmary of Edinburgh, Edinburgh, United Kingdom; 5 Environment Agency, National Monitoring, Starcross, Exeter, United Kingdom; 6 International Centre of Excellence for Aquatic Animal Health, Cefas, Weymouth, United Kingdom; 7 Centre for Sustainable Aquaculture Futures, University of Exeter, Exeter, United Kingdom; 8 Analytics & Data Science Directorate, UK Health Security Agency, London, United Kingdom; University of Tennessee at Knoxville, Knoxville, Tennessee, USA

**Keywords:** wastewater, sequencing, metagenomics, SARS-CoV-2, virus, epidemiology, hybrid capture

## Abstract

Wastewater-based epidemiology is a powerful tool for monitoring the emergence and spread of viral pathogens at the population scale. Typical polymerase chain reaction (PCR)-based methods of quantitative and genomic monitoring of viruses in wastewater provide high sensitivity and specificity. However, these methods are limited to the surveillance of target viruses in a single assay and require prior knowledge of the target genome(s). Metagenomic sequencing methods may represent a target-agnostic approach to viral wastewater monitoring, allowing for the detection of a broad range of target viruses, including potentially novel and emerging pathogens. In this study, targeted and untargeted metagenomic sequencing methods were compared with tiled-PCR sequencing for the detection and genotyping of viral pathogens in wastewater samples. Deep shotgun metagenomic sequencing was unable to generate sufficient genome coverage of human pathogenic viruses for robust genomic epidemiology, with samples dominated by bacteria. Hybrid-capture enrichment of shotgun libraries for respiratory viruses led to significant increases in genome coverage for a range of targets. Tiled-PCR sequencing led to further improvements in genome coverage compared to hybrid capture for severe acute respiratory syndrome coronavirus 2, enterovirus D68, norovirus GII, and human adenovirus F41 in wastewater samples. In conclusion, untargeted shotgun sequencing was unsuitable for genomic monitoring of the low virus concentrations in wastewater samples analyzed in this study. Hybrid-capture enrichment represented a viable method for simultaneous genomic epidemiology of a range of viral pathogens, while tiled-PCR sequencing provided the optimal genome coverage for individual viruses with the minimum sequencing depth.

**IMPORTANCE:**

Most public health initiatives that monitor viruses in wastewater have utilized quantitative polymerase chain reaction (PCR) and whole genome PCR sequencing, mirroring techniques used for viral epidemiology in individuals. These techniques require prior knowledge of the target viral genome and are limited to monitoring individual or small groups of viruses. Metagenomic sequencing may offer an alternative strategy for monitoring a broad spectrum of viruses in wastewater, including novel and emerging pathogens. In this study, while amplicon sequencing gave high viral genome coverage, untargeted shotgun sequencing of total nucleic acid samples was unable to detect human pathogenic viruses with enough sensitivity for use in genomic epidemiology. Enrichment of shotgun libraries for respiratory viruses using hybrid-capture technology provided genotypic information on a range of viruses simultaneously, indicating strong potential for wastewater surveillance. This type of targeted metagenomics could be used for monitoring diverse targets, such as pathogens or antimicrobial resistance genes, in environmental samples.

## INTRODUCTION

Wastewater-based epidemiology (WBE) has emerged as a promising tool for population-scale monitoring of pathogenic organisms in human populations (1–3). WBE enables surveillance of pathogens present in the population without requiring invasive testing of individuals. It is able to sample from both asymptomatic and symptomatic individuals and from a broad range of communities, making it both a cost-effective and relatively unbiased method of data collection to inform public health decision-making (4). Most notably, WBE has been used for tracking the prevalence and spread of severe acute respiratory syndrome coronavirus 2 (SARS-CoV-2) during the coronavirus disease 2019 (COVID-19) pandemic, as well as for identifying outbreaks of poliovirus, including vaccine-derived poliovirus, outside its endemic areas since its eradication across much of the globe (1, 5, 6). However, studies have also provided proof-of-concept for surveillance of a range of other viral pathogens (7–12), as well as the surveillance of antimicrobial resistance (AMR) genes in bacterial populations (13–17).

WBE, implemented through detection and quantification by quantitative plymerase chain reaction (qPCR), can provide data on the prevalence of viral pathogens in the population (11, 18, 19) and has demonstrated the ability to identify outbreaks prior to detection in clinical samples (8, 20–22). This was augmented using genomic surveillance in wastewater during the COVID-19 pandemic to track the emergence and spread of SARS-CoV-2 variants and understand transmission patterns in the population (23–27). Due to the low concentration of SARS-CoV-2 in wastewater samples, genomic surveillance has oriented toward PCR amplicon sequencing, either by targeting specific genomic regions (28, 29) or through a tiled-PCR amplification approach across the whole genome (30), the latter being the most common method used for sequencing of clinical samples. Whole genome sequencing (WGS) via PCR can provide high sensitivity for viral detection in degraded samples, such as those from wastewater, targeting broader regions than those amplified during qPCR (31, 32).

However, targeted amplicon sequencing has thus far only been developed for monitoring individual viral pathogens. Early detection of viral outbreaks and newly emerging viral pathogens could benefit from rapid, multi-species monitoring using a target-agnostic metagenomic approach, forgoing the need for prior knowledge of viral genomes for primer and probe design. However, although human pathogenic viruses have been detected in wastewater by untargeted metagenomics, the sewage virome is dominated by bacteriophages and plant viruses (33, 34). Hybrid-capture enrichment of metagenomic libraries has been shown to be effective for the investigation of viruses in clinical samples and wastewater, significantly enhancing sensitivity in the presence of highly complex sample backgrounds (35–37). With the design of a probe panel containing species of interest for WBE, this method could provide an efficient means of monitoring a spectrum of human pathogenic viruses simultaneously.

Viruses that predominantly display fecal-oral transmission and/or are shed in high quantities in the feces of infected individuals represent good candidates for WBE. This includes a range of nonenveloped enteric viruses, which have been studied in municipal wastewater, including polioviruses, noroviruses, enteroviruses, adenoviruses, hepatitis A virus (HAV), and rotaviruses (5, 8, 9, 38, 39). Human pathogenic enveloped viruses that represent the causal agents of many recent epidemics and pandemics, such as influenza, SARS, Middle East respiratory syndrome, and Ebola viruses, were previously thought to be highly susceptible to degradation in aqueous environments (40). However, many enveloped viruses have since been shown to survive long periods in wastewater (40, 41) and be detectable in the feces of infected individuals (42–45) and sewage (7, 40, 46). This has led to the application of WBE to respiratory viruses previously thought to be unsuitable for environmental monitoring (10, 11, 47). Genomic wastewater surveillance may therefore be plausible for a broad range of human pathogenic viruses, using similar methods to those deployed during the SARS-CoV-2 pandemic.

In this study, we present a comparison of methods for both metagenomic and targeted wastewater surveillance of human pathogenic viruses using samples collected by the UK Health Security Agency Environmental Monitoring for Health Protection (EMHP) program during the SARS-CoV-2 pandemic from October 2021 to March 2022 in London, UK (2), coinciding with the transition in the predominance of circulating Delta to Omicron variants (48) and the lifting of domestic restrictions in England on 24 February 2022 (49). We compare genomic viral wastewater surveillance by untargeted shotgun deep-sequencing with hybrid-capture enrichment using a human respiratory virus probe panel, as well as WGS of a selection of human pathogenic viruses by targeted PCR amplification, including SARS-CoV-2 and poliovirus. Furthermore, we present novel primer schemes for tiled-PCR amplification of the genomes of enterovirus D68 (EV-D68), norovirus GII (NoVGII), human adenovirus (HAdV)-F41 (HAdV41), HAV, influenza A virus (IAV), and measles morbillivirus (MeV). These findings provide an insight into the relative efficacy of these genomic methods to inform future implementation of WBE for viral pathogen monitoring.

## RESULTS

### Shotgun metagenomics

**FIG 1:**
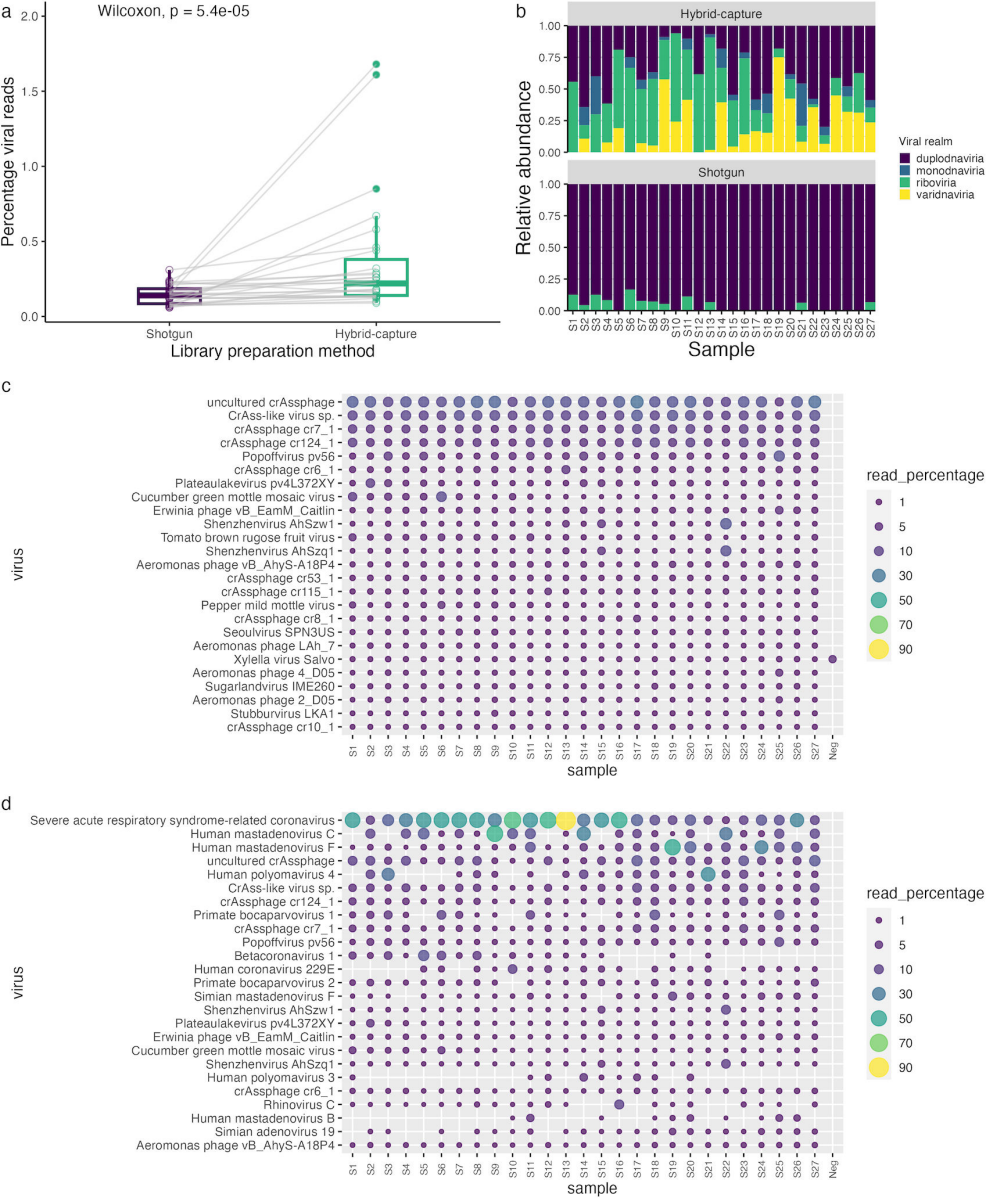
Taxonomic assignment of shotgun and hybrid-capture-enriched metagenomic reads. Plots showing the percentage of reads assigned to viruses (a) and the relative abundance of viral realms (b) across shotgun and hybrid-capture-enriched libraries. Percentage of virus-assigned reads allocated to the top 25 viruses identified in shotgun (c) and Respiratory Virus Oligo Panel hybrid-capture-enriched (d) libraries, calculated by the mean percentage of reads assigned to each taxa across the samples.

To assess the efficacy of metagenomic sequencing to detect human pathogenic viruses in sewage, total nucleic acid samples extracted from influent wastewater were sequenced using a shotgun approach. A mean sequencing depth of 303 million (SD = 9.6 million) 2× 150 bp read pairs was obtained for shotgun libraries (Table S1). Despite the extraction process, including centrifugation to remove solids and bacterial cells and ammonium sulfate precipitation to enrich for viral particles in wastewater, <0.6% of reads were assigned to viruses across all shotgun libraries (Fig. 1a), with a mean of 99.6% (SD = 0.5%) reads assigned to bacteria. Viral reads in shotgun samples were largely dominated by the virus realm Duplodnaviria (Fig. 1b), the majority of which were from bacteriophages (Fig. 1c), while the small proportion of viruses from Riboviria were dominated by plant RNA viruses (Fig. 1b and c).

### Hybrid-capture enrichment

Metagenomic libraries were also subjected to hybrid-capture enrichment using the Respiratory Virus Oligos Panel (RVOP) to investigate the utility of this method to improve coverage of a range of target respiratory viruses in metagenomic sequencing. A mean sequencing depth of 106 million (SD = 3.4 million) 2× 150 bp read pairs was obtained for hybrid-capture-enriched libraries (Table S1). The percentage of viral reads was significantly increased by hybrid-capture enrichment (Fig. 1a), while the negative control contained no viral reads. Hybrid capture also increased the diversity of viral taxa in sequencing data (Fig. 1b). This included an enhanced proportion of the Riboviria (Fig. 1b), representing an increased abundance of reads assigned to the human RNA viruses targeted by the RVOP, predominantly SARS-CoV-2 (Fig. 1d). Also enriched by hybrid capture were the viral taxa Varidnaviria and Monodnaviria (Fig. 1b), representing an increase in the abundance of adenoviruses and polyomaviruses/bocaparvoviruses, respectively, which are targeted by RVOP enrichment (Fig. 1d). Furthermore, along with HAdV-B and -C included in the RVOP, HAdV-F showed high abundance in hybrid-capture-enriched samples (Fig. 1d), suggesting cross-reactivity of these probes for other adenoviruses. These results indicate effective enrichment for target viruses in wastewater metagenomic sequencing libraries by hybrid capture.

**FIG 2:**
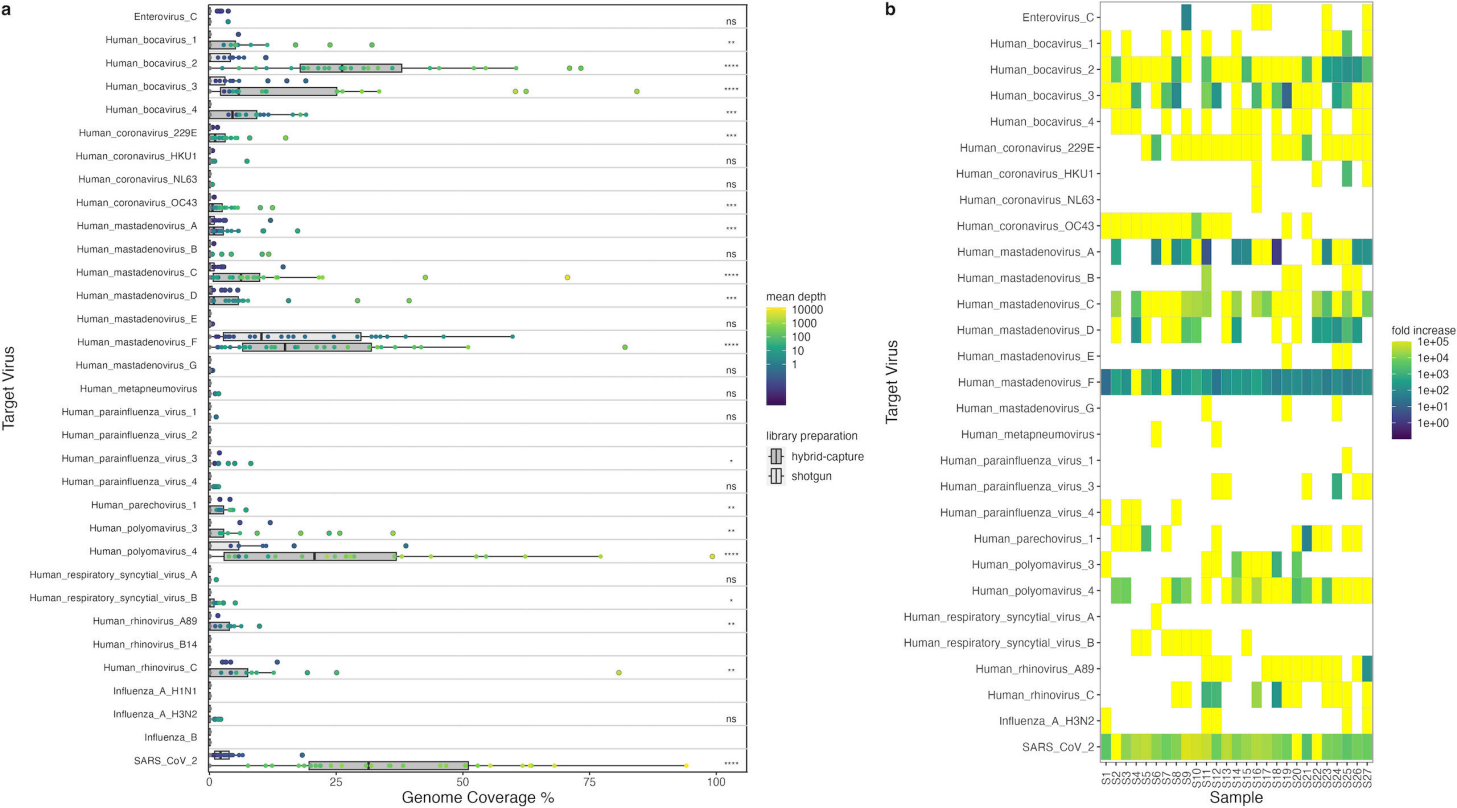
Hybrid-capture enrichment improves genome coverage and increases the sensitivity of metagenomic sequencing of viruses in wastewater. (a) Genome coverage obtained for viruses targeted by the Respiratory Virus Oligo Panel from sequencing of shotgun and hybrid-capture-enriched libraries. Genome coverage breadth is displayed in boxplots, and mean coverage depth across the target genome is represented in the color scale of points. Significance values resulting from a paired Wilcoxon signed-rank test between shotgun and hybrid-capture samples are displayed (ns, *P* > 0.05; *, *P* ≤ 0.05; **, *P* ≤ 0.01; ***, *P* ≤ 0.001; ****, *P* ≤ 0.0001), with values missing where no reads were aligned. (b) Fold increase in the percentage of reads aligned to each target virus between shotgun and hybrid-capture-enriched libraries for each sample. Yellow boxes indicate that no reads are aligned to the virus in the shotgun library.

The genome coverage of viruses targeted by the RVOP was investigated to evaluate the feasibility of the genomic epidemiology of pathogenic viruses using hybrid-capture enrichment. While three of the target viruses (human parainfluenza virus 2/human rhinovirus B14/influenza B) were not identified in either shotgun or hybrid-capture libraries, reads aligned to the remaining 25 viruses targeted by the RVOP panel, 15 of which showed a significant increase in genome coverage with hybrid-capture enrichment (Fig. 2a). Additionally, HAdV-A, -D, and -F showed significant increases in genome coverage in hybrid-capture-enriched libraries compared to shotgun libraries (Fig. 2a), providing further evidence for the cross-reactivity of the HAdV-B, -C, and -E probes in the RVOP panel. Over 50% genome coverage was obtained from hybrid-capture enrichment of some samples for human bocavirus 2 and 3, HAdV-C and -F, human polyomavirus 4, human rhinovirus C, and SARS-CoV-2, while over 25% coverage was obtained in samples for human bocavirus 1, HAdV-D, and human polyomavirus 3 (Fig. 2a).

Furthermore, the fold increase in the percentage of reads aligned to each target virus was investigated to assess the sensitivity of enrichment by hybrid capture. Many target viruses with reads identified in hybrid-capture-enriched libraries were assigned no reads in shotgun libraries (Fig. 2b), despite an approximately threefold higher sequencing depth of shotgun compared to hybrid-capture-enriched libraries. Where reads from target viruses were identified in shotgun libraries, hybrid-capture enrichment led to a fold increase in the percentage of aligned reads of >100× in 86% of cases and >1,000× in 56% of cases (Fig. 2b). Moreover, when SARS-CoV-2 reads were identified in shotgun libraries, this fold increase was between 3,000 and 97,000× (Fig. 2b). This is reflected in the increase in mean coverage depth for viruses targeted by the RVOP panel across the samples compared to shotgun libraries (Fig. 2a). Together, these findings demonstrate the high sensitivity of hybrid-capture enrichment to improve coverage of virus genomes through metagenomic sequencing of wastewater.

**FIG 3:**
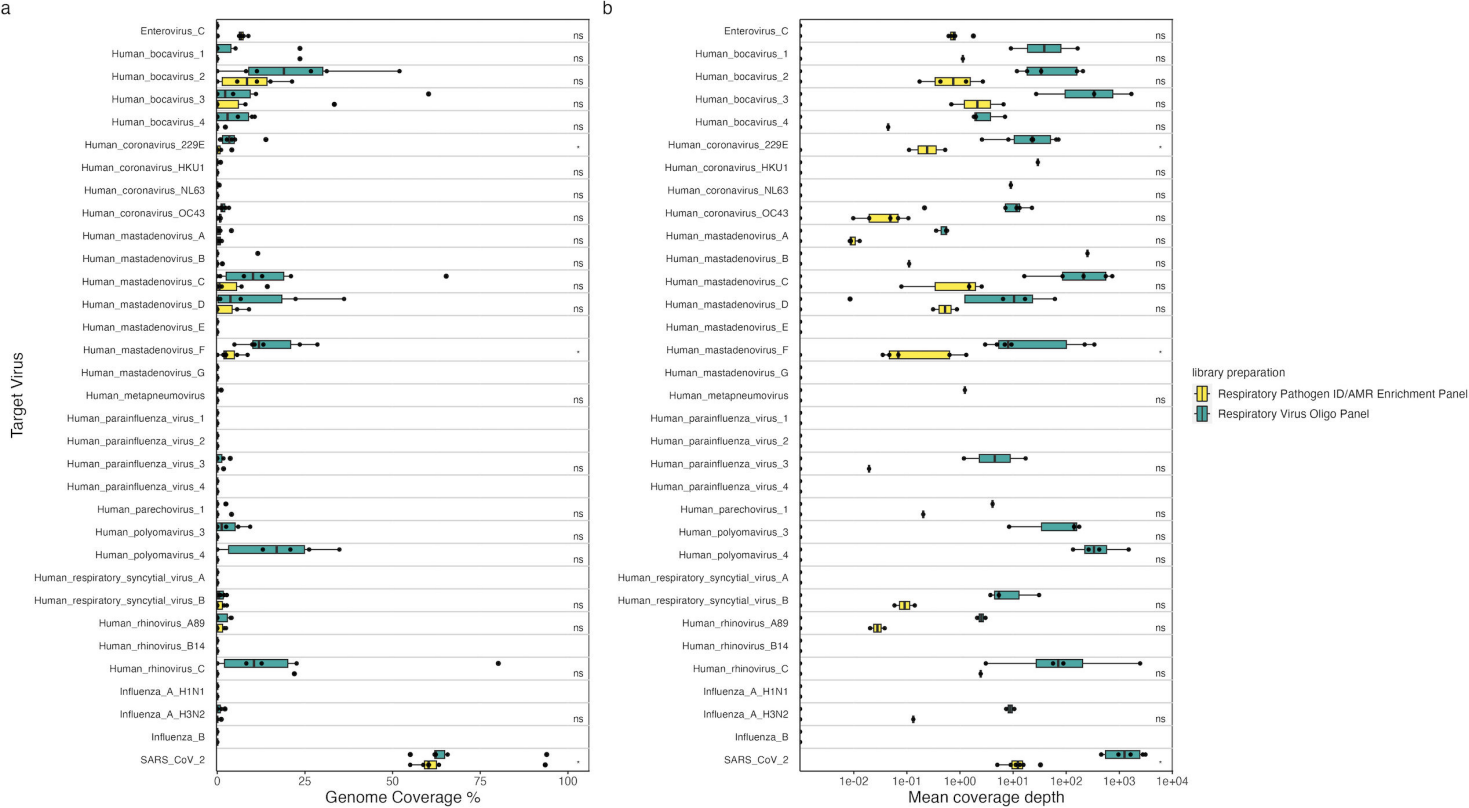
Targeting AMR genes in addition to respiratory viruses by hybrid capture reduces the sensitivity to detect viruses in wastewater (a). Genome coverage breadth (a) and depth (b) obtained for viruses targeted by the RVOP and RPIP panels. Significance values resulting from a paired Wilcoxon signed-rank test between RVOP and RPIP libraries are displayed (ns, *P* > 0.05; *, *P* ≤ 0.05), with values missing where no reads were aligned.

We also wanted to investigate whether increasing the number of hybrid-capture targets impacts the sensitivity to detect viruses. To achieve this, parallel library preparation of six samples was carried out with the RVOP as well as the Respiratory Pathogen ID/AMR Enrichment Panel (RPIP), containing the same respiratory virus probes along with a broad range of probes targeting antimicrobial resistance genes. The breadth and depth of coverage were lower across the majority of target viruses when using the RPIP panel than the RVOP panel, with this difference being statistically significant for SARS-CoV-2, HAdV-F, and human coronavirus 229E (Fig. 3). Meanwhile, the number of reads aligned to an AMR gene database was significantly increased, with a fold change between 140 and 700× in all samples (Fig. S1). The reduced coverage of viral targets suggests that increasing the number of nucleic acid species targeted by hybrid capture from the inclusion of AMR genes leads to reduced sensitivity to enrichment for viral targets in wastewater samples.

### Tiled-PCR amplicon sequencing

**FIG 4:**
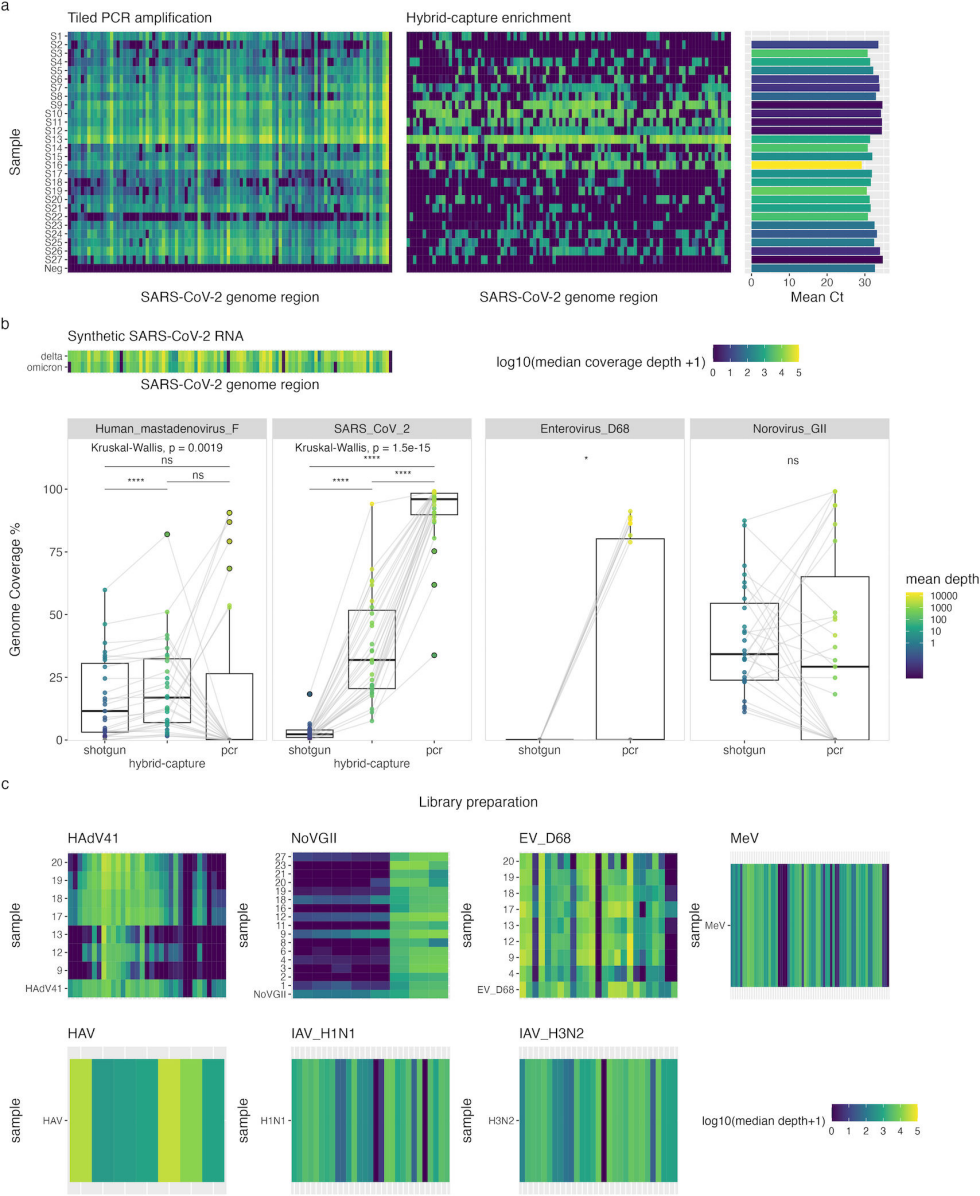
Comparison of genome coverage and variant calling results from hybrid-capture enrichment and tiled-PCR amplicon sequencing. (a) Heatmap of median coverage depth across 100 subregions (~300 bp) of the SARS-CoV-2 genome from tiled-PCR and hybrid-capture enrichment sequencing of wastewater samples and tiled-PCR sequencing of synthetic SARS-CoV-2 RNA variants Delta (B.1.617.2) and Omicron (BA.1). (b) Genome coverage obtained for target viruses from sequencing of shotgun, hybrid-capture-enriched and tiled-PCR libraries. Genome coverage breadth is displayed in boxplots, and mean coverage depth across the target genome is represented in the color scale of points. Significance values resulting from a paired Wilcoxon signed-rank test between shotgun and hybrid-capture samples are displayed (ns, *P* > 0.05; *, *P* ≤ 0.05; ***, *P* ≤ 0.0001), and the *P*-value from the Kruskal–Wallis test across categories is given for HAdV41 and SARS-CoV-2. (c) Median coverage depth across all amplicon inserts in samples, which showed successful amplification of each target virus and positive controls (MeV, HAV, NoVGII, HAdV41, EV_D68, IAV_H1N1, IAV_H3N2).

In addition to using a hybrid-capture enrichment approach for targeted sequencing of viruses in wastewater, a tiled-PCR approach was applied to the same samples for sequencing a range of target viruses. This included SARS-CoV-2, using a commercially available kit, and EV-D68, NoVGII, HAdV41, HAV, and MeV using custom-designed primer schemes. As SARS-CoV-2 was highly prevalent across the sample collection area and period (26), this provided a good example for the comparison of these methods for genomic wastewater surveillance. To support this, RT-qPCR identified SARS-CoV-2 in all samples, with a mean Ct of 32.5 (SD = 1.5; Fig. 4a). Between 370,000 and 3,500,000, 2 × 150 bp read pairs were obtained across the samples for SARS-CoV-2 amplicon libraries (Table S1). High genome coverage breadth was obtained for SARS-CoV-2 across all samples with tiled-PCR amplification (Fig. 4a), which was significantly correlated with the Ct values from RT-qPCR (Fig. S2a). SARS-CoV-2 coverage breadth from tiled PCR was, as expected, significantly greater than both shotgun and hybrid-capture enrichment libraries across the samples (Fig. 4b). In contrast, the genome coverage breadth obtained for hybrid-capture enrichment was considerably more variable (Fig. 4a and b), likely dependent on the variable concentration of SARS-CoV-2 RNA in the samples and the limits of the sensitivity of this technique. Indeed, SARS-CoV-2 coverage breadth from hybrid capture was strongly correlated with Ct values from RT-qCPR (Fig. S2b) and coverage breadth from tiled PCR (Fig. S2c). Genome coverage over 50% was observed for eight samples through hybrid capture, including one sample with 94% coverage (Fig. 4b). This demonstrates the potential of SARS-CoV-2 probes in the RVOP panel for whole-genome coverage as well as the sensitivity of tiled PCR for whole genome sequencing of SARS-CoV-2 in wastewater.

Alongside SARS-CoV-2, HAdV41 was also targeted through tiled-PCR sequencing as well as being enriched for by the RVOP panel, despite only containing probes targeting other adenoviruses. HAdV-F was identified in hybrid-capture-enriched libraries for all samples, reads from which gave significantly higher genome coverage breadth than shotgun libraries (Fig. 4b). Although only seven samples showed successful PCR amplification of HAdV41, all of these had a genome coverage breadth of above 50%, which exceeded the coverage obtained by hybrid-capture enrichment for the same sample in all cases (Fig. 4b). The low success rate of HadV41 tiled PCR was likely due to DNA degradation in the wastewater samples, reducing the chance of amplification of 1.2 kb fragments in the amplicon scheme designed for this target. Despite this, these results support the findings of improved genome coverage achieved using tiled-PCR amplification for SARS-CoV-2.

NoVGII was successfully amplified from 16/27 wastewater samples, despite being identified in shotgun libraries from all samples (Fig. 4b). The reduced success rate of tiled-PCR sequencing was again likely due to sample RNA degradation diminishing the efficacy of the 1.2 kb amplicon scheme designed for NoVGII. Although there were no significant differences in the genome coverage breadths acquired compared to shotgun libraries, hybrid-capture enrichment led to a large increase in coverage depth, with between 400- and 7,000-fold increases in mean coverage depth achieved with a fraction of the sequencing depth. For most wastewater samples amplifying NoVGII, coverage depth was heavily skewed toward the 3′ end of the genome (Fig. 4c). This was less pronounced in the positive control sample, which had 99% coverage breadth (Fig. 4c), suggesting that it was not solely caused by differences in PCR efficiency between the amplicons and may represent differences in stability across the RNA genome of NoVGII. Furthermore, a mean genome coverage breadth of 86% (SD = 4%) and depth of 11,383× (SD = 4,963×) were obtained in 8/27 wastewater samples for EV-D68 through tiled-PCR sequencing (Fig. 4b). Despite the high sequencing depth of shotgun libraries, EV-D68 was not identified in any of the samples using metagenomic methods (Fig. 4b). These results demonstrated the high sensitivity of tiled PCR for sequencing viruses with very low abundance in wastewater samples.

Of the other viruses targeted by tiled-PCR sequencing, IAV, MeV, and HAV were not identified in wastewater samples, despite successful amplification of positive control material across each genome (Fig. 4c). Additionally, genomic monitoring for poliovirus using the previously described method (1) was negative for all sample pools. MeV and HAV were also absent in metagenomic libraries from all samples, suggesting that they were not present in sufficient quantities for PCR detection in the wastewater samples tested. IAV H3N2 was detected in the hybrid-capture-enriched libraries of five samples, although these showed a genome coverage of <2.2% (~300 bp; Fig. 2a) and read counts of <1,438. This suggests that IAV was both present at a very low concentration and highly fragmented in these samples, which likely explains the failure to generate amplicons from the ~600 bp PCR scheme. The HAV and MeV primer schemes generated 97% and 90% genome coverage, respectively, from the positive control, with successful amplification of 100% and 85% of target regions, respectively. IAV primer schemes resulted in 87% genome coverage for both H1N1 and H3N2, with 79% and 86% of target regions amplified, respectively.

### Virus variant classification

**FIG 5:**
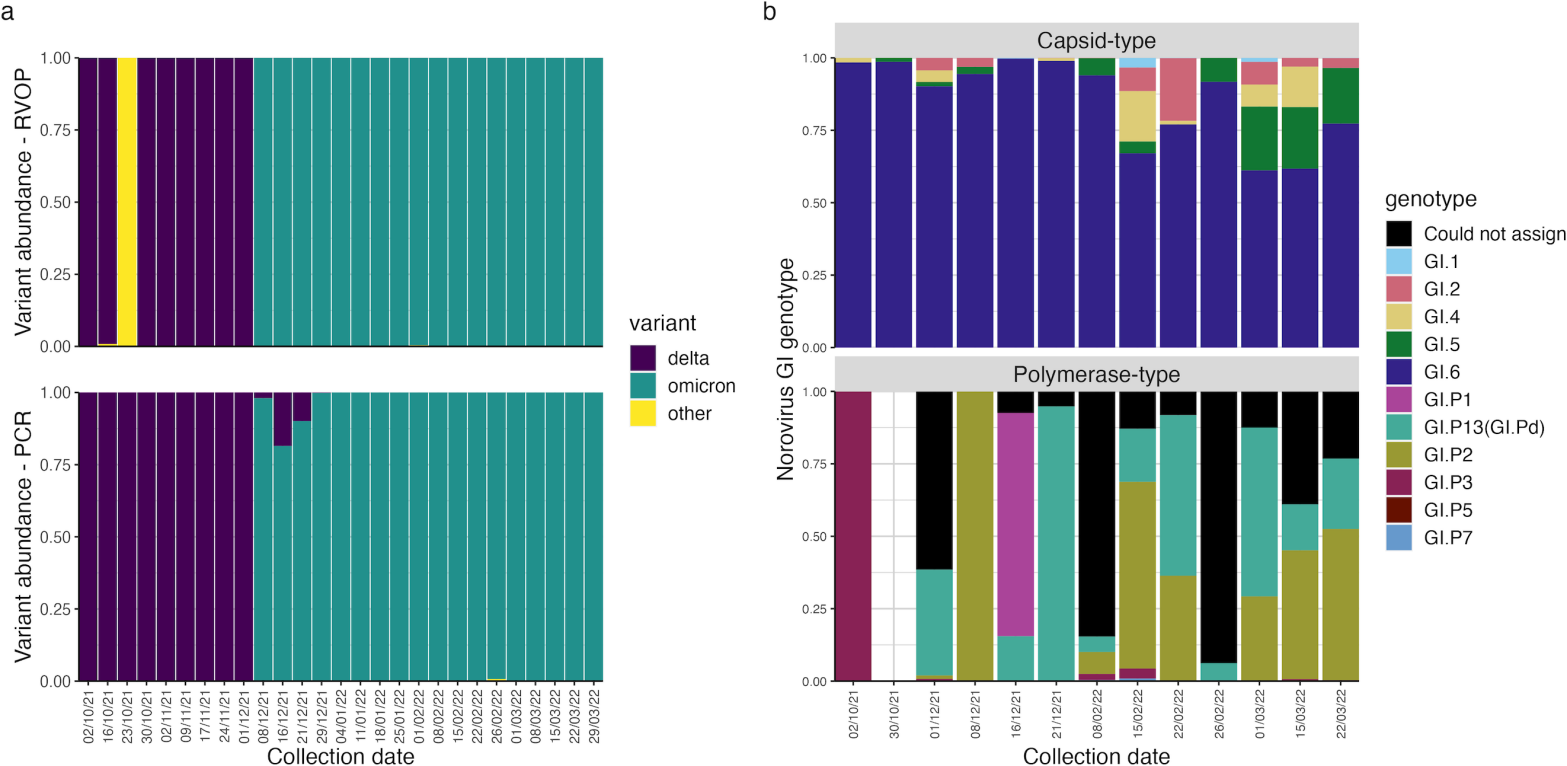
Genotyping results for PCR sequencing of SARS-CoV-2 and norovirus GI. Relative genotype abundance for (a) SARS-CoV-2, determined using Freyja, and (b) norovirus GI capsid and polymerase regions, determined using Norovirus Typing Tool (version 2.0), across each sample collection date.

To compare the utility of hybrid-capture and tiled-PCR sequencing for genotyping viruses in wastewater, lineage abundance estimation was carried out using Freyja (24) on SARS-CoV-2 alignments from these methods. Hybrid-capture results showed a switch from the Delta to the Omicron variant between samples 9 and 10, collected on 1 December 2021 and 8 December 2021, respectively (Fig. 5a). These dates are consistent with previous wastewater and individual patient testing results on the timing of the emergence of the Omicron variant in London (26), although data from amplicon sequencing in the previous study showed a more gradual increase in the relative abundance of Omicron in wastewater between late November and December 2021. The coarse resolution of variant detection using hybrid capture may be caused by the lower sensitivity of this technique, resulting in the selection of relatively few SARS-CoV-2 genomic fragments during library preparation that are then amplified in PCR steps.

Tiled-PCR data from the pooled wastewater samples in this study showed a similar switch from the Delta to the Omicron variant between samples 9 and 10, although the subsequent samples 11 and 12 display 26% and 11% relative abundance of Delta, respectively (Fig. 5a). The discovery of 98% Omicron abundance from amplicon sequencing in sample 10 may not be representative of results across London at this time point and could be a result of the limited geographic range covered by the samples included in the pool, many of which were derived from hotel wastewater. Excluding this sample, the incremental increase in Omicron abundance from tiled-PCR sequencing observed here is consistent with previous wastewater sequencing data (26). These results suggest that the additional sensitivity provided by tiled-PCR sequencing can improve the resolution of variant detection compared to hybrid-capture enrichment in wastewater samples containing variant mixtures.

Finally, amplicon sequencing was used to identify NoVGI strains in pooled wastewater samples, targeting ~300 bp fragments of the capsid protein (VP1) region of ORF2 and the RNA-dependent RNA polymerase (RdRp) region of ORF1 used for norovirus classification (50). NoVGI was only identified in the shotgun libraries of two samples, with only 1–2 reads aligning to this virus in each. Amplicon sequencing identified NoVGI in 13/27 samples, with both targeted regions amplified in all but one sample, which did not contain the RdRp amplicon (Fig. 5b). Capsid sequences of five different genotypes were identified across the samples, with genotype GI.6 being dominant across all samples (Fig. 5b). Based on polymerase sequences present in the data set, six different genotypes were identified across the samples, although many sequences could not be assigned, possibly due to coverage of amplicon regions not containing variations required for differentiation of genotypes (Fig. 5b). Of the genotyped polymerase sequences, genotypes GI.P13 (GI.Pd) and GI.P2 dominated in most samples, although genotypes GI.P3 and GI.P1 were dominant in samples from 2 October 2021 and 16 December 2021, respectively (Fig. 5b). These results show that while surveillance of NoVGI is unlikely to be feasible in wastewater through untargeted methods, amplicon sequencing of commonly used genotyping regions can be used to monitor NoVGI variants in these samples.

## DISCUSSION

In this study, the utility of metagenomic shotgun sequencing, hybrid-capture enrichment, and targeted amplicon sequencing was compared for wastewater-based epidemiology of human pathogenic viruses in wastewater. Untargeted metagenomic sequencing theoretically provides the ability to monitor all viruses without prior knowledge of the target pathogen(s), enabling the detection of emerging viral diseases. However, despite deep sequencing of shotgun libraries with around 300 million read pairs per sample, untargeted metagenomic sequencing of wastewater in this study did not provide adequate coverage breadth and/or depth of human pathogenic viral genomes for reliable genomic surveillance applications, other than identifying the presence of specific viruses, which could be achieved by qPCR at significantly lower cost. This was largely due to the dominance of bacterial material in the extracted nucleic acids from wastewater samples used in this study and the abundance of bacteriophages and plant pathogenic viruses in the viral fraction, a pattern that is common in other metagenomic studies of sewage (33, 34, 36, 51). However, through hybrid-capture enrichment and whole genome tiled-PCR sequencing, high genome coverage was achieved for a range of human pathogenic viruses in the same wastewater samples, including SARS-CoV-2, HAdV41, EV-D68, and NoVGII.

The methods of viral concentration, purification, and nucleic acid extraction have been found to impact the concentration and detection, via sequencing, of human pathogenic viruses in wastewater (37, 51, 52). Previous metagenomic studies have used techniques including filtration, ultracentrifugation, PEG precipitation, and skimmed milk flocculation, both separately and in combination, to concentrate and purify viruses in wastewater (33, 36, 51, 53–55). The volume of wastewater processed with different methods can also impact the virome detected by metagenomic sequencing, with higher volumes used in certain concentration methods associated with increases in inhibitor concentrations (51). Furthermore, the studies used DNase and/or RNase treatment to remove extracellular and extraviral nucleic acids prior to extraction to enrich the viral fraction of wastewater nucleic acids. Indeed, extracellular DNA may explain the dominance of bacterial sequences in shotgun libraries here, despite centrifugation. However, nuclease treatment may reduce the likelihood of identifying enveloped viruses that are more vulnerable to degradation in wastewater, exposing genome fragments to nucleases (41). Indeed, only two of these studies were able to identify enveloped viral families, including enveloped DNA viruses from Poxviridae and Herpesviridae (52) and sequences from Coronaviridae viruses infecting bats (55).

Wastewater samples used in this study were processed by centrifugation to remove solids before viral enrichment by ammonium sulfate precipitation. This method was chosen as a cost-effective and time-efficient protocol for use in SARS-CoV-2 surveillance through qPCR and tiled-PCR sequencing (2), for which it has provided consistent sensitivity (19, 26, 27). Furthermore, the lack of nuclease treatment during sample processing in this protocol could increase the chances of identifying enveloped RNA viruses, such as SARS-CoV-2, MeV, and influenza, which were targeted in this study. We cannot rule out that the sample processing protocol used in this study may have influenced the sensitivity of shotgun sequencing to monitor human pathogenic viruses at usable coverage depths. Indeed, the percentage of viral reads from shotgun sequencing of wastewater in other studies did exceed that obtained here (33, 36, 53). However, one study that reported genome coverage and applied ultracentrifugation and DNase treatment before extraction obtained lower genome coverage for norovirus than the present study and did not identify any adenovirus reads by shotgun sequencing (51). This provides some support for our conclusion that untargeted metagenomic sequencing is likely to be insufficient for genomic surveillance of human pathogenic viruses in wastewater, unless viral concentration can be enhanced further.

Another factor that has been found to impact the concentration of viral nucleic acids in sewage is the type of sample taken. There is increasing evidence to suggest that enveloped viruses, including SARS-CoV-2 and IAV, partition preferably to the settled solids compared to the influent wastewater (11, 22, 41, 56), while surveillance of other respiratory viruses, including metapneumovirus, parainfluenza, respiratory syncytial virus, rhinovirus, and seasonal coronaviruses, by qPCR has been demonstrated in wastewater solids (10, 47). Furthermore, one metagenomic study of sewage sludge samples identified relatively high abundances of herpesvirus and coronavirus species across the samples, while norovirus and enteroviruses were detected only by targeted PCR in all and most samples, respectively (34). Although hybrid-capture enrichment and tiled PCR were successful in generating adequate genome coverage for SARS-CoV-2 genotyping, coverage of SARS-CoV-2 from shotgun libraries was poor, and all methods failed to detect significant amounts of IAV in all samples. Considering this, future studies should perform a similar comparison of untargeted and target-enriched methods to those presented in this study with the solid fraction of wastewater samples to investigate whether genomic surveillance for respiratory viruses could be improved using this sample type.

Aside from SARS-CoV-2, which was known to be prevalent during the sampling period of this study (26), other human pathogenic viruses, such as HAdV41, EV-D68, and NoVGII, showed promising coverage from whole genome tiled amplicon sequencing in this study. Samples amplifying HAdV41 showed similar genome coverage to another study monitoring this virus in wastewater using tiled PCR (12). Viruses in the Adenoviridae family are commonly found in metagenomic sequencing of wastewater, with HAdV41 having been identified as the most common HAdV genotype here and in recent studies (51, 57). Furthermore, HAdV41 was found to be prevalent in children with acute hepatitis in early 2022 (58). Similarly, norovirus is also frequently identified in wastewater virome studies, and the dominance of the NoVGII genotype in metagenomic libraries sequenced here reflects the finding that 90% of genotyped clinical norovirus samples in England during the 2021–2022 season were NoVGII (59). Moreover, genotype GI.6 was identified as the dominant genotype of NoVGI through targeted PCR in this study, which aligns with the dominance of this variant in clinical NoVGI cases across England (59). However, the other dominant GI.3 genotype from clinical testing was not identified here, possibly due to primer incompatibility or geographic variation. Despite not identifying MeV, HAV, and IAV through tiled PCR in this study, these primer schemes show potential for use in genomic surveillance for these viruses with minor primer alterations and optimization of primer concentration balancing.

Although whole genome PCR was able to recover high genome coverage for NoVGII and HAdV41 for some samples, many samples failed to amplify these viruses despite their identification across all shotgun libraries. This is unlikely to be solely caused by PCR inhibition, considering successful amplification of SARS-CoV-2 from all samples, but may have been caused by nucleic acid degradation in wastewater samples, which can restrict the contiguous binding of primer pairs to DNA/cDNA fragments (31). This is particularly likely for the larger ~1,200 bp primer schemes used in this study for NoVGII, HAdV41, and HAV, compared to the SARS-CoV-2 amplicons, which have a mean insert size of 226 bp. Although another study using an ~1,200 bp HAdV41 amplicon scheme produced successful amplification on all five wastewater samples tested, these samples were selected from a total of 144, possibly for their high qPCR-determined HAdV41 concentration (12). A previous comparison of tiled-PCR sequencing methods for SARS-CoV-2 wastewater monitoring found that genome coverage for samples with high Ct values improved when using ~400 bp compared to ~1,200 bp amplicons (30). However, smaller amplicon schemes for larger viral genomes, such as the 34 kb genome of HAdV41, may require more optimization due to the increased chance of primer interactions with higher primer pool complexity (30). These findings together provide indications of factors to consider when designing tiled-PCR amplicon schemes for wastewater surveillance.

Hybrid-capture enrichment using a human respiratory virus probe panel led to a significant improvement in genome coverage breadth and depth for a range of viruses compared to untargeted libraries in the present study. In particular, high genome coverage was obtained from some samples for SARS-CoV-2, HAdV, rhinovirus C, and human bocaviruses. One previous study using the same hybrid-capture panel for sequencing SARS-CoV-2 in wastewater obtained complete consensus genomes for samples with Ct values <33, while sequencing without enrichment yielded a maximum of 40 read pairs (37). Moreover, another study that performed hybrid-capture sequencing on wastewater, using a probe panel targeting 207 taxa containing vertebrate viruses (35), saw significant increases in genome coverage for a range of human pathogenic viruses compared to unenriched libraries, in some cases achieving >60% genome coverage for viruses not detected without enrichment (36).

Together, these findings provide evidence for the utility of hybrid-capture enrichment for genomic surveillance of multiple pathogenic viruses. This technique provides the benefits of metagenomic sequencing through the ability to detect the emergence or introduction of uncommon viruses into a population while also monitoring the prevalence and genotype of commonly observed viral pathogens. Any viruses of particular interest that are detected could then be investigated through targeted PCR sequencing. The range of targets could also be extended beyond viruses, for example, to monitor AMR genes in wastewater bacterial populations, which have been demonstrated to reflect clinical antibiotic use (17, 60). However, although simultaneous hybrid capture for viruses and AMR genes in this study led to successful enrichment of both target sets, the sensitivity of viral enrichment was reduced, which may hinder the efficacy of viral genotyping through decreased genome coverage. This is likely caused by the relatively high concentration of bacterial material in the wastewater samples used in this study compared to viral genomic material, leading to probe-bound AMR gene fragments out-competing viral fragments during magnetic bead enrichment. However, expanding the number of targets of similar concentration in wastewater samples, such as increasing the targeted viral species, could enhance the value of this broad-spectrum technique.

Furthermore, the longer probes used in hybrid-capture enrichment (80-mer oligos in the case of the RVOP panel) are likely to be more robust to variation in target virus genomes than the short primer regions used in tiled PCR. The spread of hybrid-capture probes across the target virus genome can also be more robust to degraded samples such as those from wastewater compared to qPCR assays, which only target short genomic regions, similarly to whole genome tiled-PCR sequencing (31). However, the lower sensitivity of hybrid capture than PCR techniques increases the sequencing depth required to achieve sufficient genome coverage for genomic surveillance and reduces the limit of detection.

### Conclusions

In this study, untargeted metagenomic sequencing of wastewater did not provide sufficient genome coverage of human pathogenic viruses for robust genomic epidemiology, despite the high sequencing depth used in this study. However, using hybrid capture to enrich for a range of respiratory viruses in the same metagenomic libraries led to a significant improvement in the genome coverage obtained for these targets. This demonstrates the potential for this technique to be used for genomic surveillance of multiple pathogens of interest simultaneously, although this still requires prior knowledge of the targeted viral genomes. Whole genome tiled-PCR sequencing resulted in further improvements in genome coverage for SARS-CoV-2, HAdV41, NoVGII, and EV-D68 from wastewater samples while requiring significantly less sequencing depth, reinforcing the evidence to support this as the optimum method for genomic epidemiology of specific viruses in wastewater.

## MATERIALS AND METHODS

### Wastewater sample collection processing

Wastewater sample collection, processing, and nucleic acid extraction were carried out by the EMHP program in England (2). Briefly, 1 L of wastewater samples was collected from 42 locations across the sewer network in London, UK, between 2 October 2021 and 29 March 2022, as well as eight hotels used as quarantine sites close to Heathrow Airport, London, UK, between 8 and 16 December 2021. Wastewater was transported, stored at 4°C–6°C, and processed within 24 h of collection. Viral enrichment and nucleic acid extraction methods have been described previously in full (61). Briefly, samples were centrifuged to remove suspended solids before viral enrichment by ammonium sulfate precipitation and total nucleic acid extraction using NucliSENS magnetic extraction reagents (bioMérieux, UK) on the Kingfisher Flex purification system (ThermoFisher, UK). Nucleic acid samples were initially used for SARS-CoV-2 surveillance before being stored at −80°C until further processing.

Nucleic acid samples from each collection week were combined to form 27 pools, containing samples from between 7 and 16 sites per pool (Table S2), providing sufficient sample volume for comparison of methods while maintaining the temporal variable. The volume of each pooled sample was variable due to previous SARS-CoV-2 surveillance. Accordingly, samples 6, 17, 18, and 19 were diluted with nuclease-free water (NFW) to provide sufficient 330 µL volume for further processing (Table S2). A negative control of nuclease-free water was included in this stage and subjected to all downstream processes. A 30 µL aliquot was separated for poliovirus surveillance, while the remaining 300 µL was purified via a 1.8× clean-up with Mag-Bind TotalPure NGS (Omega Bio-tek) magnetic beads before nucleic acids were eluted in 164 µL NFW. A 16 µL aliquot of nucleic acid was separated for targeted sequencing of the double-stranded DNA virus HAdV41. A 96 µL aliquot was used for cDNA synthesis in 6× 20 µL reactions using LunaScript RT SuperMix Kit (New England Biolabs, UK), which were subsequently pooled and used for targeted sequencing of SARS-CoV-2, EV-D68, NoVGI, NoVGII, HAV, IAV, and MeV. Purified nucleic acid pools were also used for RT-qPCR quantification of SARS-CoV-2 using the previously described method (61). The remaining purified nucleic acid was used in shotgun and hybrid-capture enrichment sequencing.

### Shotgun and hybrid-capture enrichment sequencing

**FIG 6:**
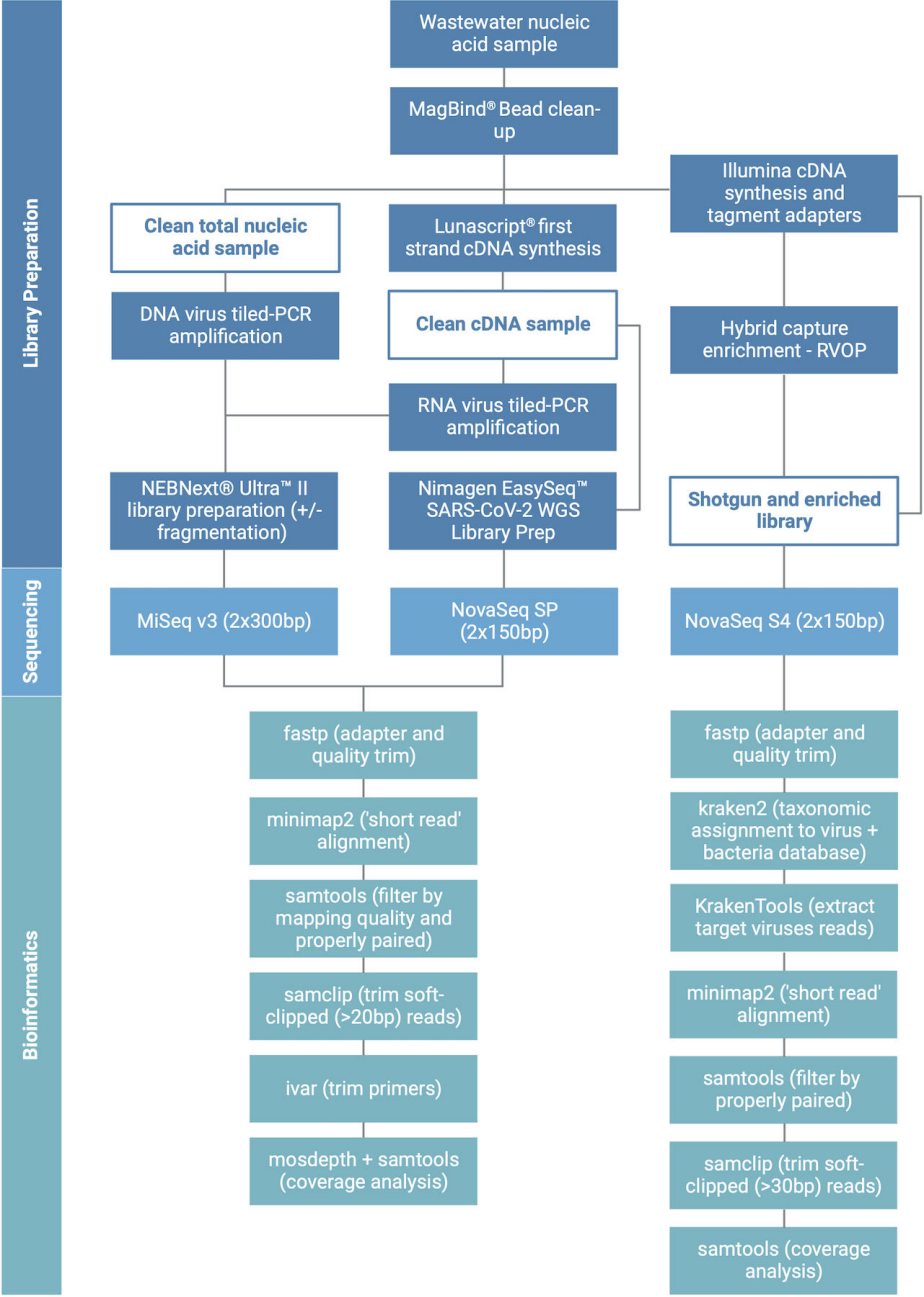
Flow diagram of the methods used in this study, including library preparation, sequencing, and bioinformatics. Created with BioRender.com.

The library preparation methods used in this study are summarized in Fig. 6. Shotgun libraries were prepared using the Illumina RNA Prep with Enrichment (L) Tagmentation Kit (Illumina, UK), following the manufacturer’s protocol. Sample RNA concentration was determined using a Qubit fluorometer using the RNA HS (High Sensitivity) Assay Kit (ThermoFisher Scientific), and library preparation input was normalized to 100 ng of RNA. Shotgun libraries were separated after clean-up of the tagmented library, before hybrid-capture one-plex enrichment was carried out using the RVOP V2 (Illumina, UK), following the manufacturer’s protocol. The hybrid capture of six shotgun libraries was repeated using the RVOP and RPIP in parallel. Shotgun and enriched libraries were normalized into pools and sequenced at 2 × 150 bp on a NovaSeq 6000 using a 300-cycle S4 Reagent Kit v1.5 (Illumina, UK).

### Targeted amplicon sequencing

A variety of PCR amplification approaches were taken to detect and identify the different viruses under investigation by targeted amplicon sequencing, depending on the diversity of each viral genome and the informative value of potential sequence reads. SARS-CoV-2 was sequenced using the EasySeq RC-PCR SAR-CoV-2 (novel coronavirus) Whole Genome Sequencing Kit (NimaGen, The Netherlands), following the previously described protocol (32), with sequencing carried out on a NovaSeq 6000 using a 300-cycle SP Reagent Kit v1.5 (Illumina, UK). Genomic surveillance for poliovirus was carried out as described previously (1), with sequencing carried out on an Oxford Nanopore Technologies MinION Mk1B sequencer using the R9.4 flow cell.

For EVD-68, NoVGII, HAdV41, HAV, MeV, and IAV, primers for tiled amplification across the whole genome were designed, while specific regions of the genome typically used for viral genotyping were amplified for NoVGI using previously published primers (62). Details of the primer scheme design for each targeted virus, positive controls used for validation, and PCR cycling conditions are provided in the supplemental methods, with primer sequences in Table S3. PCR products targeting these viruses were assessed for successful amplification by agarose gel electrophoresis. PCR products for samples displaying amplification at the expected size were taken forward for library preparation in order to improve sequencing depth for positive samples by reducing sequencing of off-target amplicons. NEBNext Ultra II DNA PCR-free Library Prep Kit for Illumina (New England Biolobs, UK) was used for samples that did not require fragmentation, including MeV, EV-D68, and NoV GI. NEBNext Ultra II FS DNA Library Prep Kit for Illumina (New England Biolobs, UK) was used for samples that required fragmentation, including NoV GII, HAdV, HAV, and IAV, following the manufacturer’s protocol with fragmentation carried out for 7 min. The resulting libraries were pooled and sequenced at 2 × 300 bp on an Illumina MiSeq using the MiSeq Reagent Kit v3 600-cycles (Illumina, UK).

### Bioinformatics analysis

The bioinformatics pipelines used in this study are summarized in Fig. 6. All Illumina reads were trimmed using fastp v0.23.1 (63) to remove sequencing adapters and low-quality bases (-q 20), and resulting reads less than half the maximum length were removed. Reads from shotgun and hybrid-capture-enriched libraries were then taxonomically assigned using kraken2 v2.1.2 (64) to a virus and bacteria database built on 4 June 2022. Proportions of reads assigned to specific taxa were extracted using pavian v1.0 (65). To investigate the genome coverage obtained through metagenomic sequencing methods, reads assigned to all viruses targeted by the RVOP panel and tiled PCR in this study were extracted using KrakenTools v1.2 extract_kraken_reads.py (66). These reads were then aligned to a concatenated fasta file containing reference genomes for each targeted virus using minimap2 v2.15 in sr mode (67). Alignments were then filtered for properly paired reads using samtools v1.15 (68), before removing reads with >30 bp soft-clipped using samclip v0.4.0 (https://github.com/tseemann/samclip). Genome coverage for each viral genome was determined using samtools coverage (68). Reads from the comparison of RVOP and RPIP hybrid-capture panels were additionally aligned to the ResFinder database (69) using minimap2, and reads aligned to each AMR gene were calculated using CoverM v0.6.1 (https://github.com/wwood/CoverM).

Trimmed reads from targeted PCR sequencing were aligned to their respective viral reference genomes using minimap2 (reference accessions in the supplemental methods). The resulting alignments were filtered to select properly paired reads with a mapping quality above 55 using samtools, before removing all reads with soft-clipped regions >20 bp using samclip to ensure that only sequences from the specific target virus were carried forward. Primer regions were then trimmed using ivar (70), before genome coverage was determined using samtools coverage and mosdepth v0.3.3 (71).

For each sample collection date, relative SARS-CoV-2 lineage abundances were estimated for the trimmed targeted PCR and RVOP reads using Freyja v1.3 (24) with a curated lineage file and UShER global phylogenetic tree downloaded on 4 February 2022. The NoVGI amplicon sequences obtained for each sample collection date were genotyped by analyzing the capsid and polymerase regions using the Norovirus Typing Tool v2.0 (50).

## Data Availability

All sequencing data files are available from the European Nucleotide Archive (ENA) database (PRJEB62830).

## SUPPLEMENTAL MATERIAL

Figure S1 mbio.01468-23-s0001.tif Comparison of the number of reads aligned to the ResFinder antimicrobial resistance gene database from sequencing of six wastewater nucleic acid pools with hybrid-capture enrichment using the Respiratory Virus Oligo Panel (RVOP) and the Respiratory Pathogen ID/AMR Enrichment Panel (RPIP) probe panels. Gray lines connect read counts from the same sample, and the *P*-value from the Wilcoxon matched-pairs signed-rank test is given.

Figure S2 mbio.01468-23-s0002.tif Correlation between SARS-CoV-2 genome coverage and Ct value from RT-qPCR quantification of SARS-CoV-2 for wastewater pools sequenced with (a) tiled-PCR amplification and (b) hybrid-capture enrichment with the Respiratory Virus Oligo Panel (RVOP), and (c) correlation between SARS-CoV-2 genome coverage from both of these sequencing techniques. Pearson correlation coefficient (*R*) and *P*-values are given.

Supplemental text mbio.01468-23-s0003.docx Supplemental methods.

Table S1 mbio.01468-23-s0004.xlsx Raw read pair counts and summary statistics for each sample and sequencing method.

Table S2 mbio.01468-23-s0005.xlsx Collection timestamps, sample IDs, and SARS-CoV-2 Ct values for samples contained in each pool.

Table S3 mbio.01468-23-s0006.xlsx Primer sequences used in this study.

ASM does not own the copyrights to Supplemental Material that may be linked to, or accessed through, an article. The authors have granted ASM a non-exclusive, world-wide license to publish the Supplemental Material files. Please contact the corresponding author directly for reuse.
