# Erratum for Child et al., “Comparison of metagenomic and targeted methods for sequencing human pathogenic viruses from wastewater”

**DOI:** 10.1128/mbio.02558-24

**Published:** 2024-08-30

**Authors:** Harry T. Child, George Airey, Daniel M. Maloney, Abby Parker, Jonathan Wild, Suzie McGinley, Nicholas Evens, Jonathan Porter, Kate Templeton, Steve Paterson, Ronny van Aerle, Matthew J. Wade, Aaron R. Jeffries, Irene Bassano

## ERRATUM

Volume 14, no. 6, e01468-23, 2023, https://doi.org/10.1128/mbio.01468-23. Page 19: Reference 62 should appear as follows.

Ollivier J, Lowther J, Desdouits M, Schaeffer J, Wacrenier C, Oude Munnink BB, Besnard A, Mota Batista F, Stapleton T, Schultz AC, Aarestrup F, Koopmans M, de Graaf M, Le Guyader S. 2022. Application of next generation sequencing on norovirus-contaminated oyster samples. EFSA Supporting Publications 19(6):EN-7348. https://doi.org/10.2903/sp.efsa.2022.EN-7348

