# Supplementary figures and images for: Comparison of metagenomic and targeted methods for sequencing human pathogenic viruses from wastewater
Source: mBio. 2023 Oct 25;14(6):e01468-23. doi: 10.1128/mbio.01468-23 (PMC10746264; doi:10.1128/mbio.01468-23)

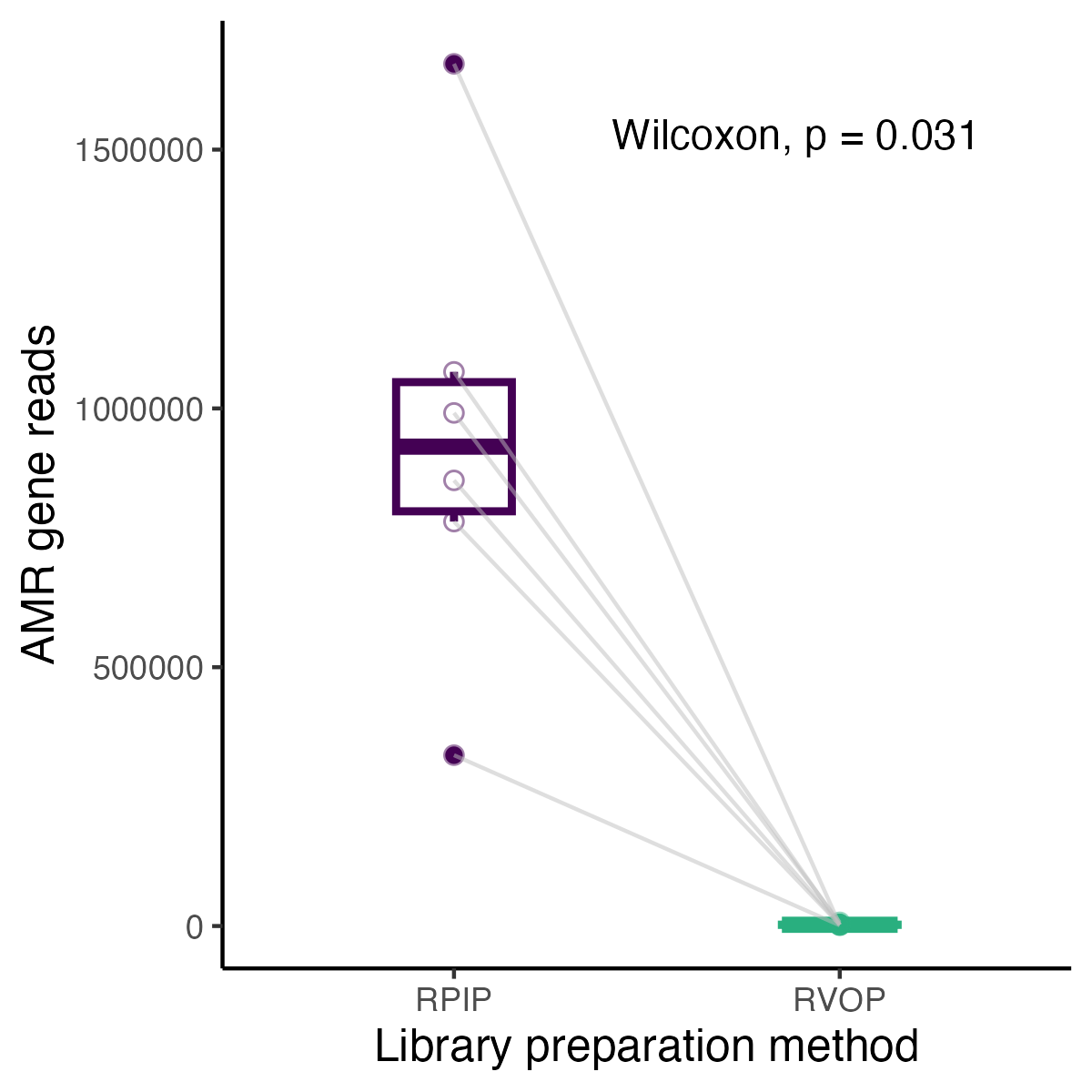

Supplement: Figure S1 — Comparison of the number of reads aligned to the ResFinder antimicrobial resistance gene database from sequencing of six wastewater nucleic acid pools with hybrid-capture enrichment using the Respiratory Virus Oligo Panel (RVOP) and the Respiratory Pathogen ID/AMR Enrichment Panel (RPIP) probe panels. Gray lines connect read counts from the same sample, and the P-value from the Wilcoxon matched-pairs signed-rank test is given. [file mbio.01468-23-s0001.tif]

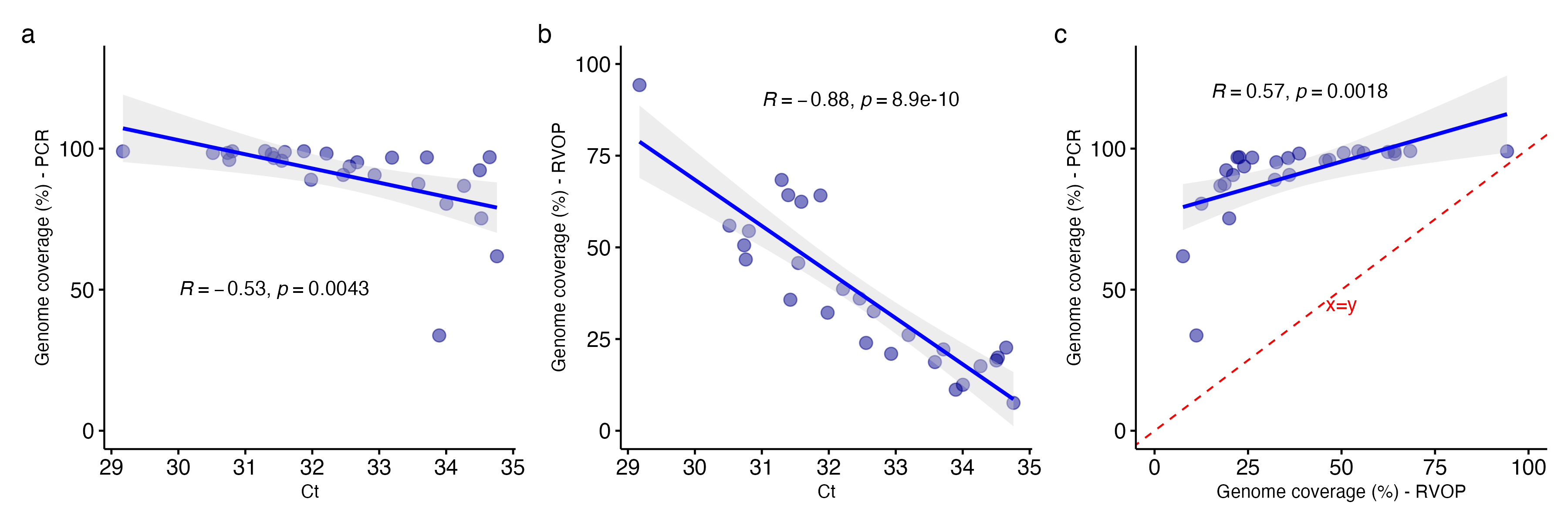

Supplement: Figure S2 — Correlation between SARS-CoV-2 genome coverage and Ct value from RT-qPCR quantification of SARS-CoV-2 for wastewater pools sequenced with (a) tiled-PCR amplification and (b) hybrid-capture enrichment with the Respiratory Virus Oligo Panel (RVOP), and (c) correlation between SARS-CoV-2 genome coverage from both of these sequencing techniques. Pearson correlation coefficient (R) and P-values are given. [file mbio.01468-23-s0002.tif]
