# Supplementary material for: Comparison of metagenomic and targeted methods for sequencing human pathogenic viruses from wastewater
Source: mBio. 2023 Oct 25;14(6):e01468-23. doi: 10.1128/mbio.01468-23 (PMC10746264; doi:10.1128/mbio.01468-23)
Supplement: Supplemental text — Supplemental methods. [file mbio.01468-23-s0003.docx]

**Supplemental Methods**

Presented here are the methods used to design primer schemes for amplicon sequencing of target viruses in wastewater samples, as well as details of PCR conditions and positive control material used for validation. Also provided are the reference genomes used in alignments for all viruses targeted by PCR and hybrid-capture enrichment.

**Enterovirus D68 (EV-D68**)

Primers for tiled-amplification of the EV-D68 genome were developed using the software MEGA and PrimalScheme. Eight whole-genome references (GenBank: NC_038308, MT791934, MT789751, MT789750, MT789749, KY767821, KT764078, KT280501) were concatenated from the Virus Pathogen Database Analysis Resource (ViPR; <https://www.viprbrc.org/brc/home.spg?decorator=vipr)>. All references are from virus isolated from human hosts in Western Europe between 2014 and 2018. PrimalScheme generated 25 primer pairs spanning the approx. 7.3kb genome in 400bp amplicons. Due to the genetic similarity of different enterovirus species, and the number of primers incorporated in this scheme, these primers are cross-reactive with a number of other viral species (Human poliovirus 1, Coxsackievirus A19, A20, A21, and Enterovirus C99) according to NCBI BLAST analysis of percentage identity of primer sequences. Positive PCR reactions therefore hold the potential to result from a number of different enteroviruses. Synthetic EV-D68 RNA was obtained from Twist Biosciences (UK) for use as a positive control.

**PCR cycle used for EV-D68 amplification**

| Temp (^o^C) | Time | Cycles |
| --- | --- | --- |
| 98 | 30 secs | 1 |
| 98 | 10 secs | 30 |
| 46 | 20 secs |  |
| 72 | 20 secs - 2 mins |  |
| 72 | 2 mins | 1 |

**Norovirus GII (NoVGII)**

Sixteen whole-genome norovirus genogroup two (NoVGII) references (GenBank: NC_039477, MH218636, MH218633, MH218617, MH218613, MH218715, MH218708, MH218706, MH218704, MH218700, MH218698, MH218695) were downloaded from ViPR, isolated from human hosts in the twenty first century, and aligned on MEGA. Sequence diversity was too great for a tiling scheme to be produced by PrimalScheme, or for NoVGII to be tiled at approximately 400bp. Therefore, an approximately 1.2kb amplicon scheme was manually developed to tile the 7.5kb genome, with the incorporation of degenerate bases to complement all references. This method resulted in eight primer pairs. These amplicons were fragmented during library preparation for Illumina sequencing platforms. Positive control RNA originated from Public Health England Culture Collections Lenticule discs RNA extractions.

**PCR cycle used for NoV GII amplification**

| Temp (^o^C) | Time | Cycles |
| --- | --- | --- |
| 98 | 30 secs | 1 |
| 98 | 10 secs | 16 |
| 55 – 0.5/cycle | 20 secs |  |
| 72 | 2 mins |  |
| 98 | 10 secs | 14 |
| 40 + 0.2/cycle | 20 secs |  |
| 72 | 2 mins |  |
| 72 | 5 mins |  |

**Norovirus GI (NoVGI)**

The VP1 gene encodes for the major capsid protein of noroviruses, which undergoes significant levels of sequence mutation and recombination making it a strong candidate for assembling phylogenetic histories, and for metabarcoding positive samples. A PCR method was chosen for sequencing the junction between ORF1 (containing RNA-dependent RNA polymerase gene) and ORF2 (containing VP1 gene) in NoVGI using previously published primers (Olivier et al., 2022). Although positive control RNA from Public Health England Culture Collections Lenticule discs RNA extractions failed to amplify either targeted region, successful amplification was achieved with pooled wastewater RNA samples.

**Human mastadenovirus F41 (HAdV41)**

A whole genome tiled-PCR scheme was developed for HAdV41 on PrimalScheme. A single HAdV41 reference genome (GenBank: DQ315364.2) was utilized from 2007. The tiling scheme divides the 34kb genome into 33 amplicons at approximately 1.2kb each. Synthetic HAdV41 RNA was obtained from Twist Biosciences (UK) for use as a positive control.

**PCR cycle used for NoV GI and HAdV amplification**

| Temp (^o^C) | Time | Cycles |
| --- | --- | --- |
| 98 | 30 secs | 1 |
| 98 | 10 secs | 30 |
| 60 | 20 secs |  |
| 72 | 30 secs |  |
| 72 | 2 mins | 1 |

**Hepatitis A virus (HAV)**

Whole genome sequences of ten HAV strains (GenBank: MN832785, MH577310, KX035096, LC435031, LC191189, MN832785, OK625565, LC515196, KF569906, MG049743) from ViPR between 2002 to 2018 were aligned on MEGA and used to generate an approximately 1.2kb amplicon scheme on PrimalScheme. Positive control material was obtained from the Centre for Environment, Fisheries and Aquaculture Science, UK, as single-stranded cDNA.

**PCR cycle used for HAV amplification**

| Temp (^o^C) | Time | Cycles |
| --- | --- | --- |
| 98 | 30 secs | 1 |
| 98 | 10 secs | 30 |
| 66 | 20 secs |  |
| 72 | 90 secs |  |
| 72 | 2 mins | 1 |

**Measles morbillivirus (MeV)**

Whole genome sequences of two MeV strains (GenBank: MG912589, MG912594) were aligned on MEGA and used to generate an approximately 400bp amplicon scheme. Synthetic MeV RNA was obtained from Twist Biosciences (UK) for use as a positive control.

**PCR cycle used for MeV amplification**

| Temp (^o^C) | Time | Cycles |
| --- | --- | --- |
| 98 | 30 secs | 1 |
| 98 | 10 secs | 30 |
| 58 | 20 secs |  |
| 72 | 25 secs |  |
| 72 | 2 mins | 1 |

**Influenza A virus (IAV)**

The IAV genome is made up of eight segments (PB2, PB1,PA, HA, NP, NA, MP, and NS) each encoding unique protein functions. Genetic reassortment of these segments can occur when a host cell is infected with multiple viruses, leading, in turn, to the evolution of new strains (Bouvier and Palese, 2008). This reassortment is of particular importance, in regard to the emergence of pandemic IAV strains, for the HA (haemagglutinin) and NA (neuraminidase) segments; viral surface glycoproteins that determine humoral immunity in hosts. Infection with influenza is seasonally-driven and cases peak in the winter months. Therefore, subtyping cases in the Southern Hemisphere winter can inform what strains are likely to affect the Northern Hemisphere in their winter, and vice-versa. Influenza surveillance in Australia in 2022 reported 217,898 notifications of laboratory-confirmed influenza. Of these, 82.1% are IAV; 94.5% of which are un-subtyped, 0.8% typed as H1N1, and 4.7% as H3N2 (Health.gov.au, accessed 2022), supporting the selection of H1N1 and H3N2 reference genomes for this investigation.

Ten IAV reference genomes were downloaded from NCBI Influenza Virus Resource Database from human hosts in the UK between 2018 and 2022 (five H1N1 and five H3N2). Each of the eight genomic segments was tiled manually at 600bp on MEGA, whereby six segments are universal for H1N1 and H3N2; and the HA and NA segments are unique for both IAV subtypes.

**PCR cycle used for IAV amplification**

| Temp (^o^C) | Time | Cycles |
| --- | --- | --- |
| 98 | 30 secs | 1 |
| 98 | 10 secs | 30 |
| 60 | 20 secs |  |
| 72 | 90 secs |  |
| 72 | 2 mins | 1 |

**Samples positive by PCR** (0- negative, 1- positive)

| Week/Date | EV-D68 | NoV GII | NoV GI | HAdV | HAV | MeV | IAVs |
| --- | --- | --- | --- | --- | --- | --- | --- |
| 1 | 0 | 1 | 1 | 0 | 0 | 0 | 0 |
| 2 | 0 | 1 | 0 | 0 | 0 | 0 | 0 |
| 3 | 0 | 1 | 0 | 0 | 0 | 0 | 0 |
| 4 | 1 | 1 | 1 | 0 | 0 | 0 | 0 |
| 5 | 0 | 0 | 0 | 0 | 0 | 0 | 0 |
| 6 | 0 | 1 | 1 | 0 | 0 | 0 | 0 |
| 7 | 0 | 0 | 0 | 0 | 0 | 0 | 0 |
| 8 | 0 | 1 | 0 | 0 | 0 | 0 | 0 |
| 9 | 1 | 1 | 1 | 1 | 0 | 0 | 0 |
| 10 | 0 | 0 | 1 | 0 | 0 | 0 | 0 |
| 11 | 0 | 1 | 1 | 0 | 0 | 0 | 0 |
| 12 | 1 | 1 | 1 | 1 | 0 | 0 | 0 |
| 13 | 0 | 0 | 0 | 1 | 0 | 0 | 0 |
| 14 | 0 | 0 | 0 | 0 | 0 | 0 | 0 |
| 15 | 0 | 0 | 0 | 0 | 0 | 0 | 0 |
| 16 | 0 | 1 | 0 | 0 | 0 | 0 | 0 |
| 17 | 1 | 0 | 0 | 1 | 0 | 0 | 0 |
| 18 | 1 | 1 | 0 | 1 | 0 | 0 | 0 |
| 19 | 1 | 1 | 1 | 1 | 0 | 0 | 0 |
| 20 | 1 | 1 | 1 | 1 | 0 | 0 | 0 |
| 21 | 0 | 1 | 1 | 0 | 0 | 0 | 0 |
| 22 | 0 | 0 | 1 | 0 | 0 | 0 | 0 |
| 23 | 0 | 1 | 1 | 0 | 0 | 0 | 0 |
| 24 | 0 | 0 | 0 | 0 | 0 | 0 | 0 |
| 25 | 0 | 0 | 1 | 0 | 0 | 0 | 0 |
| 26 | 0 | 0 | 1 | 0 | 0 | 0 | 0 |
| 27 | 0 | 1 | 0 | 0 | 0 | 0 | 0 |

**Reference genomes used in alignments**

| **virus** | **reference_accession** |
| --- | --- |
| SARS_CoV_2 | MN908947.3 |
| Influenza_A_H1N1 | NC_026438.1,NC_026435.1,NC_026437.1,NC_026433.1,  NC_026436.1,NC_026434.1,NC_026431.1,NC_026432.1 |
| Influenza_A_H3N2 | NC_007373.1,NC_007372.1,NC_007371.1,NC_007366.1,  NC_007369.1,NC_007368.1,NC_007367.1,NC_007370.1 |
| Human_coronavirus_229E | NC_002645.1 |
| Human_coronavirus_OC43 | NC_006213.1 |
| Human_coronavirus_HKU1 | NC_006577.2 |
| Human_coronavirus_NL63 | NC_005831.2 |
| Human_mastadenovirus_A | NC_001460.1 |
| Human_mastadenovirus_B | NC_011203.1 |
| Human_mastadenovirus_C | NC_001405.1 |
| Human_mastadenovirus_D | NC_010956.1 |
| Human_mastadenovirus_E | NC_003266.2 |
| Human_mastadenovirus_F41 | NC_001454.1 |
| Human_mastadenovirus_G | DQ923122.2 |
| Human_bocavirus_1 | NC_007455.1 |
| Human_bocavirus_3 | NC_012564.1 |
| Human_bocavirus_2 | NC_012042.1 |
| Human_bocavirus_4 | NC_012729.2 |
| Human_metapneumovirus | NC_039199.1 |
| Human_respiratory_syncytial_virus_A | JX627336.1 |
| Human_respiratory_syncytial_virus_B | NC_001781.1 |
| Human_polyomavirus_3 | NC_009238.1 |
| Human_polyomavirus_4 | NC_009539.1 |
| Human_parechovirus_1 | FM178558.1 |
| Human_rhinovirus_A89 | M16248.1 |
| Human_rhinovirus_B14 | NC_001490.1 |
| Human_rhinovirus_C | NC_009996.1 |
| Enterovirus_C | NC_002058.3 |
| Human_parainfluenza_virus_3 | NC_001796.2 |
| Human_parainfluenza_virus_1 | NC_003461.1 |
| Influenza_B | Influenza_B |
| Human_parainfluenza_virus_2 | NC_003443.1 |
| Human_parainfluenza_virus_4 | AB543336.1 |
| Norovirus_GI | NC_001959.2 |
| Norovirus_GII | NC_039477.1 |
| Enterovirus_D68 | NC_038308.1 |
| Measles_morbillivirus | NC_001498.1 |
| Hepatovirus_A | NC_001489.1 |
